# Tea; Its Effects, Medicinal and Moral

**Published:** 1840-01

**Authors:** 


Art. IV.—*Tea; its Effects, Medicinal and Moral.* By G. G. Sigmond, m.d., f.s.a., f.l.s., Professor of Materia Medica to the Royal Medico-Botanical Society.—*London,* 1839. 8vo, pp. 144.

The progress of civilization, in the course of which we have learned how to poison ourselves with opium, or to end its attendant happiness by hanging and drowning, has taught us to employ atmospheric pressure, by means of the stomach-pump, and the use of artificial respiration; and the same civilization, bringing with it various new and minor ills incident to humanity, has, for many of these, supplied the antidote of tea. Manifold are the opinions, both public and professional, as to the utility and wholesomeness of tea; and various the preference given to black or green. Cobbett is said to have asserted, that he never enjoyed health until he renounced the "slop kettle," and betook himself to good beer; the student constantly finds that tea is essential to any available activity of his brain; tea relieves the languor and drowsiness of the habitually heavy feeder, and somewhat diminishes the intoxicating effects of a little too much wine; washerwomen and card-playing old ladies depend on it for much of their success in life; and so do thousands besides, whose fatigues or excitements are beyond their natural or acquired capabilities of endurance, and who find in tea the real elixir vitæ, if not the vital principle itself.

The professional opinions on the propriety of tea depend on various causes, and in part, perhaps, on the doctor's own organization. The hard, phlegmatic doctor, whose nervous system was omitted in his constitution, believes that tea is a great mistake: this is the man, without imagination or reflection, who pins his faith on lecturers and books, and, because it was the fashion in the day when he had the misfortune to gain the little knowledge or ignorance which he has acquired, he still sees "diseased liver" in all human ills; and tea is "bilious" in other people's stomachs, and in his eyes. Another class approves of something more warming, and considers the appetite for drinks to have been given for a wiser purpose than to satiate it on tea; to these, the taste acquired in their students' days has grown with their growth and strengthened with their strength, and they are well acquainted with the merits of good Cognac. Their prescriptions, doubtless, abound in tinctures and essential oils. Others condemn or approve the use of tea, because they find it injurious or beneficial to themselves, without sufficiently reflecting on the circumstances which render it good or bad. There is, besides, an eclectic class, happily the largest.

It is probably correct to say, that, considering that civilized mankind is a machine generally used in a manner but little consistent with health, tea is one of the oils of its worn and rusted wheels. Had we to write a book on the moral and medicinal effects of tea, we should first endeavour to ascertain what we mean by tea. An examination of the moistened leaves of our teapots, the tea having been bought of sellers of the ordinary reputation, often reveals the fact that it is not an infusion of the tea-leaf alone which we have had the pleasure of drinking. The poorer classes constantly drink, under the name of tea, a beverage, dark, offensive, and, without a large qualifying addition of sugar, intolerable. This is frequently prepared from what is called "tea-dust," the powder of the tea-chests, with the addition of some unknown dirt. By many, also, this vegetable matter (if vegetable it be) is boiled, in order to extract from it all its virtues. Others, again, occasionally obtain the well-infused leaves, which would otherwise be employed by the housemaid in laying the dust on the floors, and we have heard of the infused leaves having been dried and re-employed. In these, and in various other ways, it is very difficult to ascertain the nature of the fluid which finds its way into the bowels of the rich and poor under the name of tea; and it is, consequently, a very difficult matter to write anything satisfactory on the subject. Had we to write a book on the moral and medicinal effects of tea, premising and making proper allowance for the difficulties and doubts above alluded to, we should endeavour to gather our information from all quarters, from the healthy and unhealthy, the rich and poor, the young and old, studious and empty-headed, idle and laborious, temperate and the reverse; and we should scarcely think we had gained much that was worthy of publication, until the information which was thus extensively derived had been judiciously and carefully prepared. Dr. Sigmond's book has entirely disappointed us. Its title is attractive, its contents the reverse. We eagerly sought for information, and, except in its extracts from other works, we found scarcely any. We were prepared for good Souchong or Pekoe, and all that was offered us was exceedingly weak Bohea. As in duty bound, we drank the Bohea, but really cannot recommend to our readers a similar draught. There was scarcely a lump of sugar in the cup.

The following remarks are worthy of attention:

"Tea, as a morning beverage, when breakfast forms a good substantial meal, upon which the powers for the day, of meeting the various changes of life, depend, provided it be not too strong, is much to be recommended; but when individuals eat little, coffee certainly supports them in a more decided manner; and, besides this, tea, without a certain quantity of solid aliment, is much more likely to influence the nervous system. Some persons, if they drink tea in the morning and coffee at night, suffer much in animal spirits and in power of enjoyment of the pleasures of society; but if they reverse the system, and take coffee in the morning and tea at night, they reap benefit from the change; for the coffee, which to them in the morning is nutrition, becomes a stimulus at night; and the tea, which acts as a diluent at night, gives nothing for support during the day." (p. 111.)

Those who suffer from the feelings termed nervousness, and who are in the habit of adding spirits to their tea for their relief, are recommended "to renounce tea altogether, and to substitute for it a beverage, half coffee, half warm milk, and, if possible, to acquire the habit of taking a substantial breakfast, which alone can dissipate this symptom of uneasiness." (p. 112.) The evil effects of green tea, on certain idiosyncrasies, are forcibly pointed out; and we believe that its effects upon the nervous system, when taken as a strong infusion, are, very generally, decided and injurious. There is some little inconsistency, in Dr. Sigmond's facts, in favour of the total abstinence from fermented liquors, and in his subsequent commendations of a little good wine or beer. For our own parts, on this subject, we doubt the majority of facts, as they are called. Did we believe them, we could not, as we heartily do, agree with the author, that "good wine is a good cordial, a fine stomachic, and, taken at its proper season, invigorates mind and body, and gives life an additional charm. There can be found no substitutes for the fermented liquors, that can enable man to sustain the mental and bodily labour which the artificial habits of society so constantly demand. . . . . If, on the one hand, disease and sorrow attend the abuse of alcoholic liquors, innocent gaiety, additional strength, and power of mind, and an increasing capability of encountering the ever-varying agitation of life, are amongst the many good effects which spring from a well-regulated diet, in which the alcoholic preparations bear their just proportions and adaptation." The argument against the use of spirits, which we have commonly found to be employed, is the argument against their abuse. And looking medically at the question, the same arguments would lead to the conviction of the propriety of total abstinence from purgatives or bleeding, or any other mode of treatment of well-ascertained efficacy.

We have not said that Dr. Sigmond's book is chiefly occupied by the natural and commercial history of tea. It is beside our purpose to enter at all into the subject, but Dr. Sigmond's account of it is well worthy of perusal.—Although we are not in the habit of alluding to theological matters in our pages, we cannot abstain from asking a question, prompted by Dr. Sigmond's curious dedication,—what are the "intellectual qualifications," which not only "give to existence its richest charm," but "promise happiness in the life to come?"

